# Tourist risk assessment of pollen allergy in tourism attractions: A case study in the Summer Palace, Beijing, China

**DOI:** 10.3389/fpubh.2022.1030066

**Published:** 2022-10-20

**Authors:** Yu Zhou, Junhu Dai, Haolong Liu, Xian Liu

**Affiliations:** ^1^Key Laboratory of Land Surface Pattern and Simulation, Institute of Geographic Sciences and Natural Resources Research, Chinese Academy of Sciences (CAS), Beijing, China; ^2^University of Chinese Academy of Sciences, Beijing, China; ^3^China-Pakistan Joint Research Center on Earth Sciences, Chinese Academy of Sciences-Higher Education Commission of Pakistan, Islamabad, Pakistan

**Keywords:** tourism attractions, pollen allergy, tourist risk assessment, green zone allergenicity index model, data mining, risk matrix, Summer Palace, climate change

## Abstract

Pollen allergy has already been an increasingly prominent ecosystem disservice in tourism attractions. However, few studies have assessed the tourist risk of pollen allergy through integrating multidisciplinary knowledge of ecology, medicine, phenology, and risk management. Basing on the conceptual framework of risk assessment proposed by UNISDR, we first established an index system of pollen-allergy risk for tourists in attractions and outlined assessment methods 18 available indexes were put forward to cover three aspects: hazard of plant allergen, tourist vulnerability, and resilience of assessment units. Subsequently, taking the Summer Palace as the case study area, we conducted a tourist risk assessment of pollen allergy. Values of nine available indexes were obtained *via* ecological investigation, phenological observation, and data mining of visitors' logs on Sina Weibo. Risk levels of spring pollen allergy for tourists in different assessment units were revealed by combining the green zone allergenicity index model and three-dimensional risk assessment matrix. The results showed that: (1) There were seven primary pollen-allergenic plants in the Summer Palace, including *Platycladus orientalis, Sabina chinensis, Salix babylonica, Pinus tabulaeformis, Populus tomentosa Carr, Morus alba L*. and *Fraxinus chinesis*, among which *Platycladus orientalis* and *Salix babylonica* were the highest allergenic. (2) Among 18 spots, tourists faced the highest risk level of pollen allergy in spring at three spots, namely the Hall of Serenity, Hall of Benevolence and Longevity, and Gallery of Literary and Prosperity. (3) The two routes of the Long Corridor and Longevity Hill scored high on the risk level. (4) Among four areas, risk levels of the Front-hill and Rear-hill areas were high. Given the increasing spatial-temporal uncertainty of pollen allergy and tourist behaviors under global warming and urbanization, the related monitoring should be strengthened in the future. Furthermore, the dynamic and improved assessment of pollen-allergy risk should be institutionalized and be integrated into the evaluation of tourism experience quality. Tourism administration should make full use of relevant assessment results and conduct more effective risk communication.

## Introduction

Pollinosis is a “national disease” around the world. It can cause allergic rhinitis, conjunctivitis, hay fever or asthma, urticaria, and allergic dermatitis, etc. Patients may suffer from shock and have a risk of sudden death, when correlated symptoms are severe. In developed countries, 20–30% of the population is allergic to pollen (1), while the prevalence rate in China is about 14.16%^1^. Moreover, there are many people in China who developed pollinosis symptoms but did not know that their symptoms belong to the pollinosis. The economic burden of pollinosis is enormous. An analysis estimated that the annual global expenditure was about 7.9 billion dollars, including direct medical expenses and indirect economic losses (2). Besides direct and indirect economic losses, intangible costs (social costs, loss of life quality) carried by pollinosis are also enormous. Therefore, pollen allergy has already attracted worldwide attention. In 1997, the WHO pointed out that pollinosis would be a prevalent disease in the twenty first century, whose treatment and prevention should be given a high priority (2).

Studies on pollen allergy came from medicine, ecology, phenology, and some other disciplines. Among them, medicine focused on the difference in the allergenicity and emissions of various pollens, the association between its incidence and the demographic characteristics of patients, and seasonality changes of airborne pollen (3). Ecology focused on the effect of the urban environment on pollen emissions (4), the allergenic species composition in different green spaces (5, 6), the allergenic potential of parks and campuses (6, 7), and interventionist approaches for allergenic environment improvement (8). The allergenic hazard of green spaces has already been assessed by using a green zone allergenicity index model, which combined multiple indexes, such as canopy height, canopy cover, allergenic potential, pollen emission and pollination duration (6, 7). Phenology paid more attention to multi-timescale variation of the allergenic flowering duration and pollen season, and relationships between these phenological indexes and different climatic factors (9, 10). In general, most studies were on a spatial scale larger than city, and allergen studies have not yet been linked to spatial-temporal behaviors of people. Moreover, few studies on pollen allergy have integrated multidisciplinary knowledge to guide the sustainable development of tourism. From the prospective of tourism, the pollen allergy risk has not been taken into account by previous studies on the tourist safety in China (11, 12), and was ignored by the existing evaluation of experience quality in tourism attractions (13, 14).

Tourism attractions play a key role in tourism and have become more and more important with the continuous enhancement of Chinese travel desire and payment capability. In 2021, the number of National A−5A level tourism attractions in China reached 14196, and their tourism revenue increased to 2.92 trillion yuan, up 31.0% over 2020 (15). Compared with the rapid increase of number and production value, the ecosystem services and functions of tourism attractions still need to be improved. The increasingly prominent pollen allergy risk is such a typical ecosystem disservice in tourism attractions. Many common woody and herbaceous plants in tourism attractions have allergenicity. The former includes *Sabina chinensis, Populus tomentosa, Fraxinus chinensis, Rhus Typhina, Syringa reticulata*, and the latter includes *Chrysanthemum morifolium* and *Brassica rapa*. Despite their undeniably benefits of the adaptability to local climatic conditions, the ease of management, and the ability to enhance the landscape, allergenic plants make tourism attractions uncomfortable seasonally for the human respiratory system. Under the background of nature tourism and outdoor education becoming more and more popular, people' exposure to the allergenic environment is increasing.

Climate change have posed unprecedented challenges to human health (16–18). As for the pollen allergy, the flowering dates of *Morus, Robinia* and *Cerasus yedoensis* across many regions have already advanced (19, 20), the pollen seasons of *Tilia sp. L., Quercus* and *Platanus* have prolonged (21–23), and the allergenicities of *Betula* and *Juniperus* have increased (22, 23). How to properly handle the pollen allergy risk, has become more and more important. A study has pointed out that the countermeasure of health risk caused by climate change contains at least two aspects: the establishment of a comprehensive assessment index system and the clarification of high-risk areas and periods (24). “*The 2021 China report of The Lancet Countdown on health and climate change: seizing the window of opportunity*” highlighted that the exposure and vulnerability of humans should be clarified, and the mapping of health risks should be conducted (25). At present, the risk assessment of pollen allergy serving the tourism industry is still blank in China. Consequently, this paper integrates multidiscipline knowledge to establish a comprehensive assessment index system of pollen-allergy risk for tourists and outlines preliminary approaches to estimate this risk. To illustrate the practical application of the assessment indexes and methods, a case study was carried out in the Summer Palace, Beijing, China. The index system of pollen-allergy risk described here can be used to guide related green-space plans of tourism attractions, to promote the tourist management, and to help pollen allergy patients to avoid environmental triggers in attractions during the allergenic pollen season.

## Materials and methods

### Conceptual framework of pollen-allergy risk assessment

Risk is defined as the possibility of a hazardous outcome or expected loss caused by the interaction between a natural or man-made disaster and it suffers (26). The United Nations International Strategy for Disaster Reduction (UNISDR) argued that risk assessment can be conducted by use of the conceptual framework of “Risk (R) = Hazard (H) × Vulnerability (V)” (27). Under this framework, the safety of landscapes and tourists in attractions has been assessed effectively (11, 28). After considering the general applicability of this framework, we used it to assess the pollen-allergy risk of tourists in tourism attractions. The assessment was conducted at three spatial hierarchies (scenic spot, route, and area), taking account of the space structure and functional partition of tourism attractions.

#### Construction of index system

The index system was made of four layers: target (A), factor (B), category index (C) and bottom index (D). The pollen-allergy risk of tourists in attractions was defined as the target (A). According to the risk conception and the pollen-allergy formation mechanism, the target was determined by three factors on the B layer, including the hazard of plant allergen, tourist vulnerability, and resilience of assessment units. As for the hazard of plant allergen, its category indexes and bottom indexes were selected considering both the biological and ecological characteristics of allergenic species. With reference to the meanings of risk exposure and the resistibility of human body in the assessment theory of natural disaster risks (29, 30), category indexes and bottom indexes of tourists vulnerability were constructed. The resilience of assessment units was designed to have no category index and the selection of its bottom indexes also referenced indexes of risk resistance capacity used in disaster-risk assessments (29, 30).

**Figure 1:**
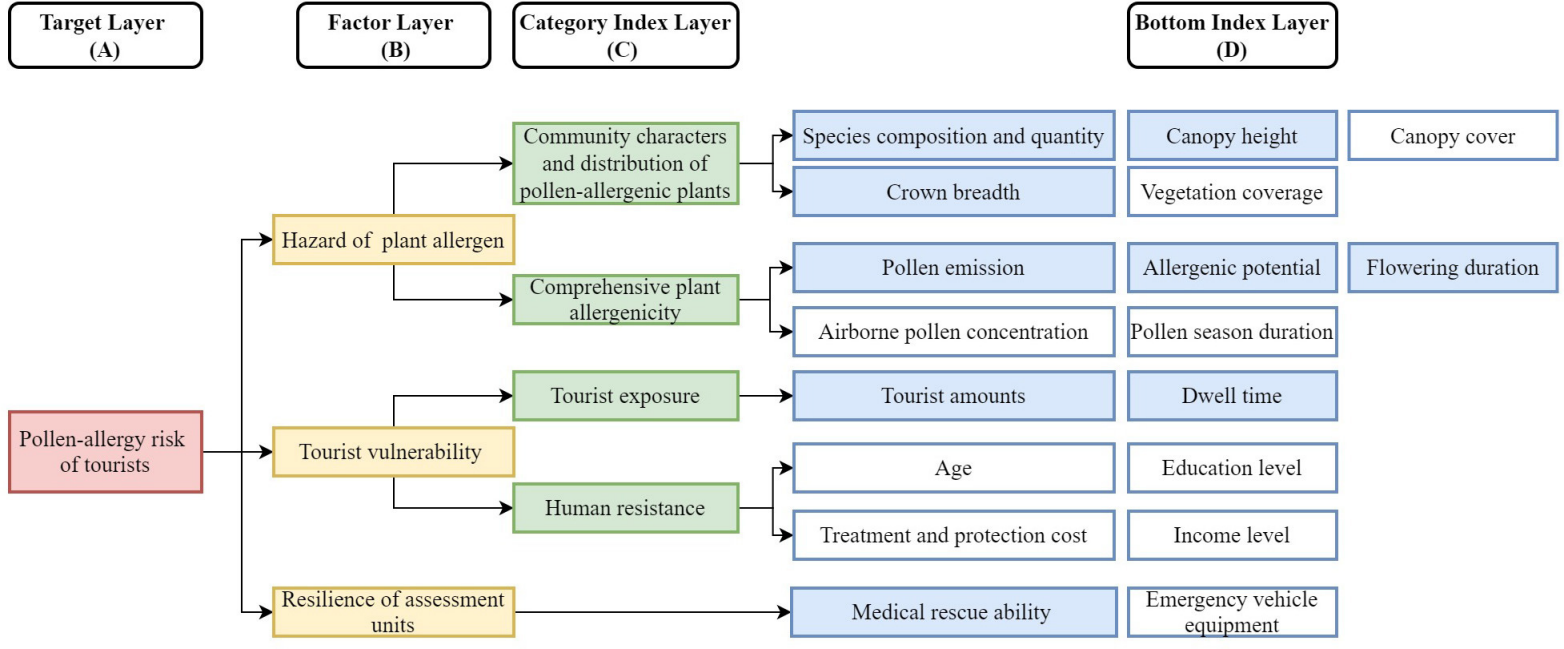
The construction of index system for tourist pollen-allergy risk in attractions (Blue indexes were used in the case study of the Summer Palace).

As shown in Figure 1, 18 bottom indexes were selected to describe different category indexes and factors. As for the first category index—community characters and distribution of allergenic plants, we selected five bottom indexes which were often used in previous ecological studies (5–7), namely the species composition and quantity, canopy height, canopy cover, crown breadth, and vegetation coverage. The values of relevant indexes can be obtained by combining the ecological investigation and remote-sensing image interpretation. There are also five commonly used indexes which can describe the second category index—comprehensive allergenicity of species: pollen emission, allergenic potential (AP), airborne pollen concentration, pollen season duration, and flowering duration (31). Their values are documented in medical and phenological reports and databases, or available *via* phenological observations and airborne allergen monitoring. Concerning the third category index of tourist exposure, we selected two basic indexes (tourist amounts and dwell time) used in most studies on tourist behaviors. More tourist amounts and dwell time mean greater tourist exposure. According to previous studies, tourist amounts can be obtained *via* the sampling survey, GPS tracking survey (32), Weibo data mining (33). Dwell time of tourists in assess units can be roughly graded into different scales through evaluating the landscape value and environmental capacity of tourism resources, or be precisely calculated by using an Agent-Based Model (ABM) (34). Regarding the fourth category index—human resistance, it is closely related to the age, education level, treatment and protection cost, income level (35, 36). If the four values can be obtained through survey questionnaire, the human resistance will be measured well. Finally, as for the resilience of assessment units, we recommended two bottom indexes (medical rescue ability and emergency vehicle equipment) with reference to disaster-risk studies. The medical rescue ability can be measured *via* calculating the shortest distance between the assessed unit and the nearest medical center on the ArcGIS platform. The numbers of equipped emergency vehicles can be obtained from the attraction's administration.

#### Quantification and coupling of assessment indexes

To eliminate the effect on data comparability imposed by dimensions and attributes, data normalization and criterion classification should be handled. There are multiple normalization techniques, such as Min-Max normalization, Z-score normalization, and Mean normalization. As for the classification criteria of indexes, most statistical values can be arranged into different “natural” classes by using the Jenks optimization method. Previous studies also provided classification criteria of some specific indexes (Table 1).

**Table 1:** Classification criteria of specific indexes for pollen allergy risk.

**Assessment indexes**	**Scale class**	**Classification criteria**
Pollen emission^a^	1	Only a few visible pollen granules on the plant are released when gently shaken or blown into the palm of the observer onto a dark surface.
	2	Many granules are released.
	3	A layer of pollen covers the palm, or a cloud of pollen can be seen in the air when the wind blows.
Allergenic potential (70)	1	Less than three sneezes per time.
	2	Intermittent nasal itching, 3–9 sneezes per time, having a running nose less than four times per day, occasional nasal congestion, intermittent eye itching.
	3	Having a tolerable sense of ant movement in the nose, continuous 10–14 sneezes per time, having a running nose 5–9 times per day, constant nasal congestion, and obvious and tolerable itching of eyes.
	4	Having an intolerable sense of ant movement in the nose, more than 15 sneezes per time, having a running nose more than 10 times per day, continuous nasal congestion, and intolerable itching of eyes.
Pollen duration or flowering duration (6)	1	Less than 1 week
	2	1–3 weeks
	3	4–6 weeks
	4	More than 6 weeks
Airborne pollen concentration	1	Woody pollen: ≤ 100; herbal pollen: ≤ 50
(granule/1000mm^2^) (37)	2	Woody pollen: 101~250; herbal pollen: 51~100
	3	Woody pollen: 251~400; herbal pollen: 101~150
	4	Woody pollen: 401~800; herbal pollen: 151~300
	5	Woody pollen: >800; herbal pollen: >300
Human Age (35, 36)	1	The elder (≥65 years old)
	2	Adolescence (≤17 years old) and the middle-aged (46–64 years old)
	3	Youth (18–45 years old)

a https://usanpn.org/files/npn/reports/USA-NPN_Plant_and_Animal_Phenophase_Definitions_v2.0.pdf.

Referring to the assessment experience of disaster risks (29, 30), there are two applicable techniques for the coupling of pollen-allergy risk indexes, namely the multi-factor model and risk matrix. As for the former, there are several approaches to determine the weight of each index, which can be divided into subjective, or objective, or combined approaches. Common subjective approaches include the Delphi method, analytic hierarchy process (AHP) and so on, which is simple but greatly affected by the subjective preferences of assessors. Objective weighting approaches without decision-maker involvement include equal weighting method, standard deviation weighting method, entropy weighting method, principal component analysis (PCA), etc. The popular Green Zone Allergenicity Index (I_GZA_) model (7, 31) (Equation 1) is essentially an equal weight method for assessing the hazard of plant allergen. Although these objective weighting approaches have a sound mathematical basis, the application of them are restricted by the amount of data and specific problem domains. Sometimes their weights did not conform to the importance of corresponding indexes. Combined approaches have the advantages of both the subjective and the objective weighting, however, their calculation process is complicated.


(1) $$\begin{array}{l} I_{GZA}=\frac{1}{378\times S_{T}}\overset{k}{\underset{i=1}{\sum }}N_{i}\times AP\times PE\times FD\times S\times H \end{array}$$

Where k = number of pollen allergenic species, N_i_ = number of individuals belonging to the i-species, AP = allergenic potential, PE = pollen emission, FD = flowering duration (d), S = vertical crown projection area covered (m^2^), H = canopy height (m), S_T_ = total surface area of the assessment unit (m^2^).

**Figure 2:**
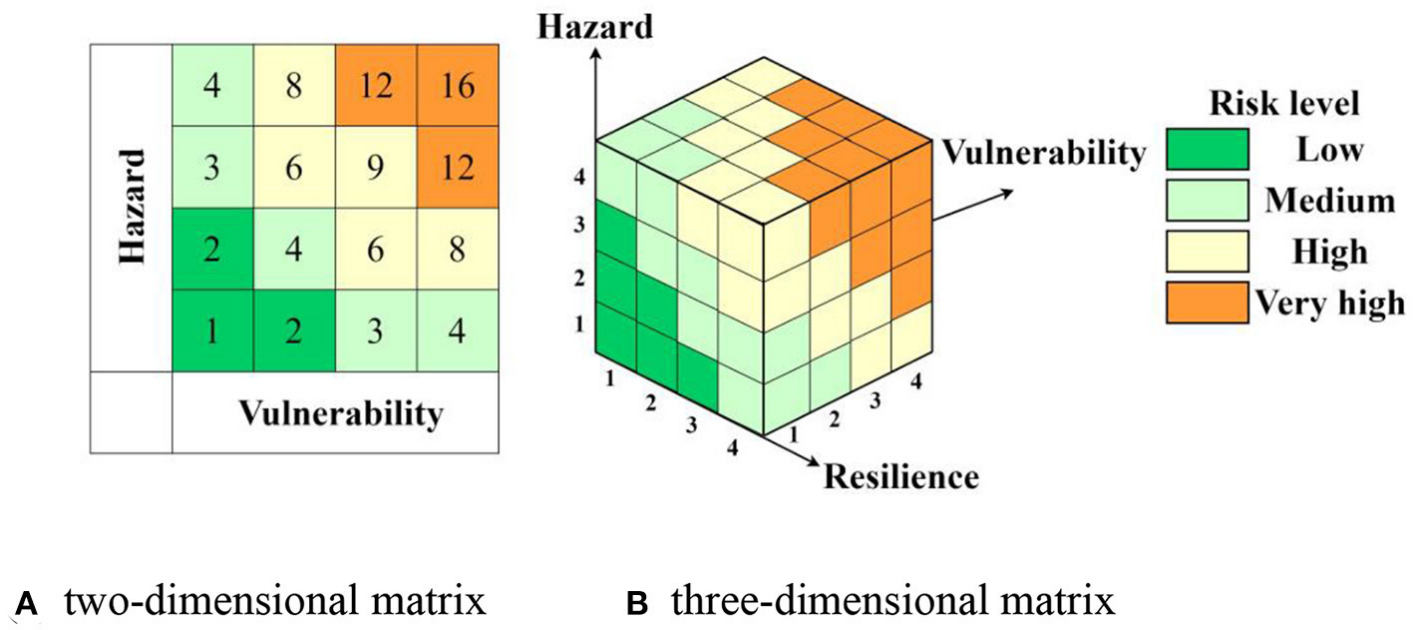
Assessment matrixes of pollen-allergy risk. **(A)** Two-dimensional matrix. **(B)** Three-dimensional matrix.

Risk level matrix is another convenient fundamental tool used to assess risk (29, 30, 38). Traditionally, the two-dimensional matrix (Figure 2A) was the most widely used. In the assessment process, a scale of 1–4 was often used, with one indicating the lowest and four indicating the highest. However, more and more experts contended that this method failed to adequately account for human factors affecting risk, and they recommended adding some human factors as a third dimension to gain a fuller picture of the true risk involved in an operation (39, 40). Therefore, according to the above-mentioned index system, we can define the three-dimensional matrix of pollen-allergy risk as a matrix between hazard, vulnerability, and resilience (Figure 2B).

### Data collection and applied model for the study area

#### Study area

Beijing, in the northwest of the North China Plain, is a typical continental monsoon region, with a semi-humid climate and deciduous broad-leaved forests. The superior geographical environment and neo-urbanization oriented by “ecological civilization” have promoted green spaces development. The present urban green space in Beijing covers an area of 926.83 km^2^, in which 357.20 km^2^ are parks (41). However, many common landscaping species in green space can cause pollinosis, including *Juniperus chinensis, Populus tomentosa, Salix babylonica, Platycladus orientalis, Pinus tabuliformis, Styphnolobium japonicum, Salix matsudana, Pinus bungeana* (5). To date, the prevalence rate of pollinosis in Beijing reached 47.02%, higher than that in other high prevalence areas in China (42).

**Figure 3:**
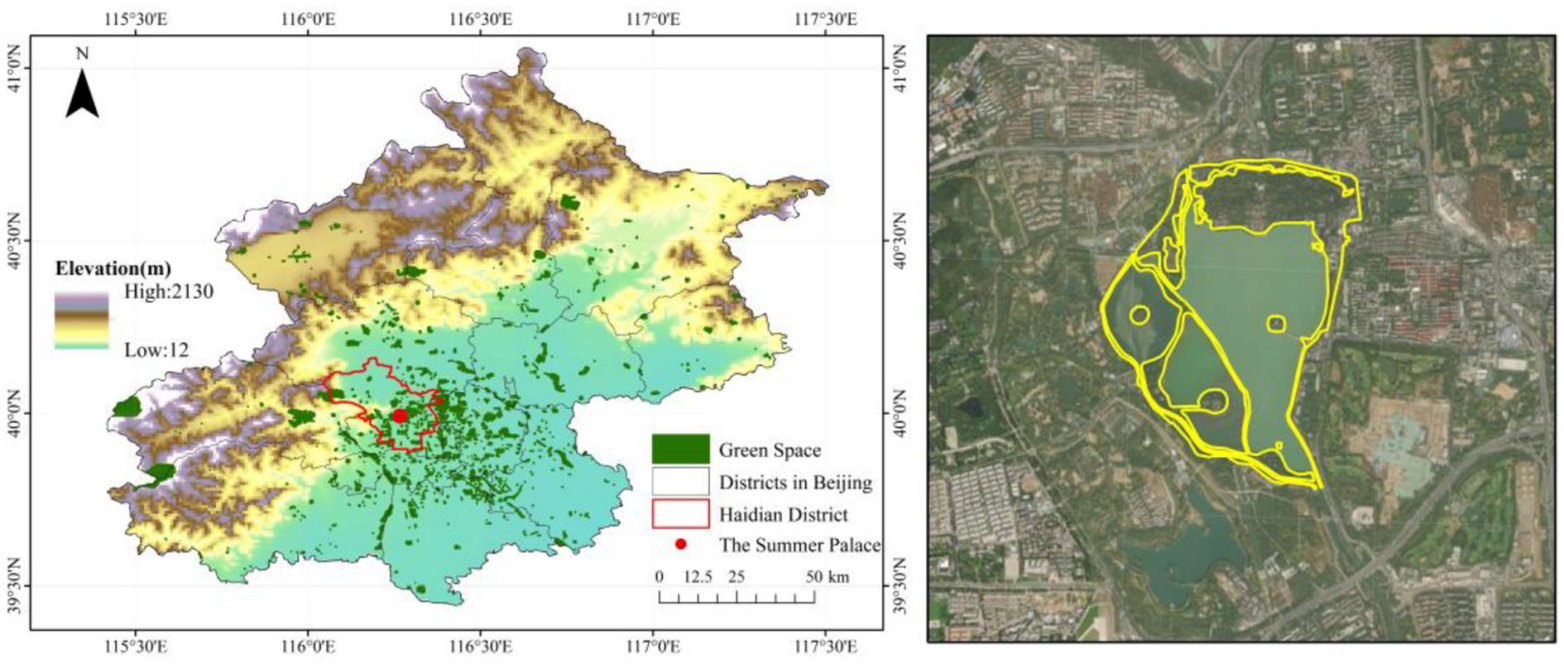
The geographical location of the Summer Palace.

The Summer Palace in the northwest of Beijing, covered an area of 290.8 hectares, in which the land area is 75.30 hectares (Figure 3). There are 22,803 pollen-allergenic trees in the park, such as *Platycladus orientalis, Sabina chinensis, and Salix babylonica*, which belong to 33 species, 11 families, and 20 genera (43). As the largest existing royal garden in China and a National 5A level tourism attraction, the average daily tourist amount of the Summer Palace exceeded 50,000 in the past decade^2^. In 2018, the park visits reached 120,000 per day during the National Day^3^, far exceeding the daily visitor capacity of 6.1 million (44). Therefore, from the perspective of plant allergen and allergen contact, the Summer Palace is a potentially high-risk area for pollen allergies.

#### Data collection

The research data used in this paper include three main categories: the first is the type and quantity data of allergenic species obtained from ecological investigation, used for addressing the distribution differences in the pollen allergen. The second is the flowering data of allergenic plants obtained through phenological observation in the Summer Palace, which was used to comprehensively analyze the hazard of pollen allergen. The third is visitors' logs data obtained by data mining of Sina Weibo, used for analyzing the difference in tourists amounts among different spots, routes and areas in the park.

##### Ecological investigation data

The ecological investigation was carried out by means of a sample plot survey in April 2021. This period was chosen because trees in April have more branches, blossoms, and stamens per unit of surface area than in other months of spring. We divided the land area in the Summer Place into different grids with a resolution of 150 × 150 m. In each grid, at least one 10 × 10 m quadrat was set. The exact geographical locations of allergenic plants within the quadrat were marked by use of the GPS. The diameter at breast height (DBH), canopy breadth and canopy height of every allergenic plant were measured by DBH ruler, tape measure, and laser rangefinder, respectively. During the investigation course, The DBH was measured at the height of 1.3 m, and the canopy breadth was calculated as described by Liu et al. (45).

##### Phenological observation data

There are seven main allergenic woody species in the Summer Place, including *Platycladus orientalis, Sabina chinensis, Salix babylonica, Pinus tabulaeformis, Populus tomentosa Carr, Morus alba L*, and *Fraxinus chinesis*. Their phenological observation data were obtained from the Chinese Phenological Observation Network (CPON, http://www.cpon.ac.cn/). Records of first flowering dates (FFD), full blooming dates (FBD) and end of flowering dates (EFD) of them in 2021 were extracted from the dataset, whose observation process conformed to the traditional criteria in China (46). According to these criteria, the FFD is the date when at least three flowers came out on the plant individual. The FBD is the date when more than half of the flowers on the tree bloomed. The EFD is the date when only 5% of the flowers remained on the plant.

##### Visitors' logs data retrieved from Sina Weibo

According to the flowering observation data in the Summer Place over the past years, there is a spring peak of local pollen amount persisting from March to May. Consequently, the index of tourist amounts was also analyzed during the same period. Social media check-in data is considered as an important data which can reflect the spatiotemporal characteristics of tourist amounts (47, 48). In light of this, between 1 March and 31 May 2021, the sign-in logs of tourists to the Summer Palace were retrieved from Sina Weibo (https://weibo.com/). By use of a search engine developed by Sichuan University (33) and the application programming interface (API) provided by Sina Weibo, we conducted the data retrieval. The settings of retrieval environment were shown in Table 2. There were 72,137 valid records after data cleansing, whose useful information included names, locations, coordinates, check-in times, tourist activities and so on. We identified 19 main points of interest (POI) in the Summer Palace. Among them, the West Causeway was treated as a scenic route because of its spatial form, and the other points were treated as scenic spots.

**Table 2:** The search settings and results of Sina Weibo.

**Search settings**			**Raw results**	**Filter settings**		**Filtered results**
**Semantics recognition**	**Year**	**Month**		**White words**	**Black words**	
Tourist behaviors	2021	March to May	75857	e.g., take photos, make videos, sightsee, and hike.	e.g., work, patrol, serve, and take a boat.	72137

#### Applied model

According to the overall tourism planning for the Summer Palace (44) and its tourism introduction on the official website, the risk assessment units in the park were ascertained as 18 scenic spots, five scenic routes and four scenic areas. Because of the limited data accessibility, we only selected nine bottom indexes to assess the tourist risk of pollen allergy in attractions, including species composition and quantity, canopy height, crown breadth, pollen emission, allergenic potential, flowering duration, tourist amounts, dwell time, and medical rescue ability.

When coupling these indexes, we firstly assessed the hazards of plant allergen in different units by use of the above-mentioned I_GZA_ model. Secondly, the tourist vulnerabilities were assessed by combination of tourist amounts and dwell time. Subsequently, the medical rescue abilities of different units were measured *via* calculating their shortest distances to the six service centers located at North Ruyi Gate, North Palace Gate, East Palace Gate, New Built Gate, South Ruyi Gate, and West Palace Gate. Values of the three risk factors were all classified into four levels *via* the natural break classification method. Finally, pollen allergy risks in different scenic spots, routes and areas were revealed by using the three-dimensional assessment matrix whose factor portfolios were shown in Table 3.

**Table 3:** Factor portfolios of the three-dimensional risk matrix (39, 40).

**Risk level**	**Factor portfolios**										
Very high	444	443	442	434	433	424	344	343	334	333	244
High	441	432	431	423	422	414	413	342	341	332	324
	323	314	243	242	234	233	224	144	143	134	
Medium	421	412	411	331	322	321	313	312	241	232	231
	223	214	213	142	141	133	132	124	123	114	
Low	311	222	221	212	211	131	122	121	113	112	111

## Results analysis

### Hazard of plant allergen in spring

Based on the phenological observation, ecological investigation and literature review, the distribution and community characteristics of seven primary pollen-allergenic trees in the Summer Palace were revealed effectively. These plants can be found throughout the park, which are densely distributed on the Longevity Mountain. According to Table 4, the numbers of *Platycladus orientalis, Sabina chinensis* and *Salix babylonica* ranks the top three. Based on the AP scale classes for different trees (7, 31, 49–51), *Fraxinus chinesis* was identified as the species with the highest allergenic potential in the Summer Palace, followed by *Platycladus orientalis* and *Sabina chinensis*. As for the pollen emissions, *Platycladus orientalis, Sabina chinensis* and *Fraxinus chinesis* were identified as the top three. The flowering durations of *Platycladus orientalis, Populus tomentosa Carr* and *Morus alba L*. all reached 20 days in 2021, while that of *Pinus tabulaeformis* was only 11 days. Overall, the allergenic hazards of *Platycladus orientalis* and *Salix babylonica* were the top two in the spring of 2021, according to the calculation results of the I_GZA_ model.

**Table 4:** List of pollen-allergenic species in the Summer Palace and their parameters.

**Specie**	**Number (N)**	**Allergenic potential (AP)**	**Pollen emission (PE)**	**Flowering duration (FD) (Unit: d)**	**Mean vertical projected area of crown (S) (Unit:m^2^)**	**Mean canopy height (H) (Unit: m)**	**Hazard level**
*Platycladus orientalis*	9446	3	3	21	13.72	9.6	4
*Sabina chinensis*	4062	3	3	16	15.20	10.1	2
*Salix babylonica*	3536	2	2	16	44.40	16.1	3
*Pinus tabulaeformis*	3205	1	2	11	95.03	13.4	2
*Populus tomentosa Carr*	682	2	2	20	94.99	17.8	2
*Morus alba L*.	196	2	1	20	67.02	14.2	1
*Fraxinus chinesis*	114	4	3	16	94.99	11.8	1

Among the 18 scenic spots in the Summer Palace, the Hall of Serenity, Garden of Harmonious Interests, Hall of Benevolence and Longevity, and Gallery of Literary Prosperity had the highest pollen-allergenic hazard in the spring of 2021. The allergenic hazards on the scenic routes of Long Corridor and Longevity Hill were higher than those on the three others. Among the four scenic areas, tourists faced higher allergenic hazard in the East-lake and Rear-hill areas (Table 4). In general, the high hazard of spring allergen in the Summer Palace was mainly distributed within the range of Longevity Mountain in 2021.

### Tourist vulnerability and unit resilience

Tourist vulnerability (namely tourist exposure in the case study) was revealed by combining the data mining of visitors' check-in on Sina Weibo and the dwell-time analysis. Among the 18 scenic spots, the Hall of Benevolence and Longevity, East Palace Gate, Gallery of Literary Prosperity and North Palace Gate were most visited in the spring of 2021. Therefore, tourist vulnerabilities of them were the highest. In contrast, the scenic spots of the Marble Boat, Chamber of Cultivation, Hall of Happiness and Longevity, Chamber of Clearness, and West Palace Gate had low tourist vulnerabilities. Among the five scenic routes, the tourist vulnerabilities of the Long Corridor route and the Longevity Hill route were very high and high, respectively, while those of the West and East Causeway routes were both low. As for the scenic areas, the tourist vulnerability of the Front-hill area was very high, while that of the East-lake area was the lowest (Table 5). Overall, tourist vulnerabilities in the north and east of the Summer Palace were higher than those in the south and west.

**Table 5:** Assessment results of pollen-allergy risk for tourists in the Summer Palace.

	**No**.	**Name**	**Hazard**	**Vulnerability**	**Resilience**	**Element portfolio**	**Risk level**
Scenic spot	A1	North Palace Gate	1	4	1	141	Medium
	A2	Suzhou Street	3	2	1	321	Medium
	A3	Hall of Serenity	4	2	4	424	Very high
	A4	Chamber of Clearness	3	1	4	314	High
	A5	Garden of Harmonious Interests	4	2	3	423	High
	A6	Chamber of Cultivation	2	1	3	213	Medium
	A7	Purple Cloud Gate Tower	2	3	2	232	Medium
	A8	Hall of Celebrating Virtues	3	3	2	332	High
	A9	Hall of Happiness and Longevity	2	1	2	212	Low
	A10	Hall of Jade Ripples	1	2	2	122	Low
	A11	Hall of Benevolence and Longevity	4	4	2	442	Very high
	A12	East Palace Gate	3	4	1	341	High
	A13	Gallery of Literary Prosperity	4	4	3	443	Very high
	A14	Marble Boat	2	1	4	214	Medium
	A15	Garden of Farming and Weaving	2	3	4	234	High
	A16	West Palace Gate	1	1	1	111	Low
	A17	South Ruyi Gate	1	2	1	121	Low
	A18	South Lake Island	1	3	3	133	Medium
Scenic route	B1	Rear-hill and back-lake route	1	2	1	121	Low
	B2	Longevity Hill route	3	3	2	332	High
	B3	Long Corridor route	4	4	3	443	Very high
	B4	East Causeway route	2	1	2	212	Low
	B5	West Causeway route	1	1	4	114	Medium
Scenic area	C1	Rear-hill area	3	3	2	332	High
	C2	Front-hill area	2	4	3	243	High
	C3	East-lake area	4	1	2	412	Medium
	C4	West-lake area	1	2	4	124	Medium

Because all the six medical centers are located near the gates of the park, the distances from the assessment unit to the nearest gate have great influence on their resilience. Among the 18 scenic spots, four scenic spots had the poorest resilience, namely the Garden of Farming and Weaving, Chamber of Clearness, Marble Boat, and Hall of Serenity (Table 5). According to the comparisons among the five scenic routes, the resilience of the West Causeway route was the poorest, while the route of the Rear-hill and back-lake was the best. The resilience of the West-lake area, Rear-hill area, the East-lake area, and Rear-hill area was decreased in turn.

### Risk difference of pollen allergy for different assessment units

**Figure 4:**
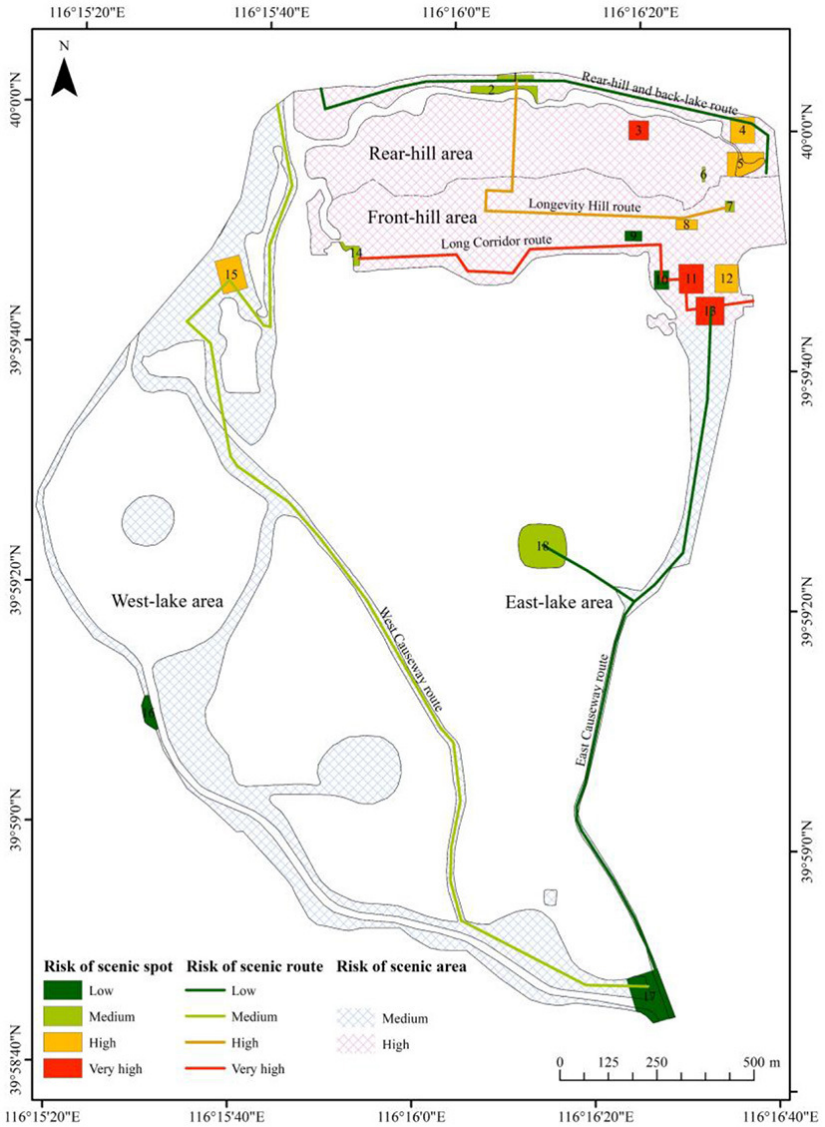
Distribution of pollen allergy risks for tourists in the Summer Palace.

According to the assessment results of the three-dimensional risk matrix, tourists in three scenic spots (namely the Hall of Serenity, Hall of Benevolence and Longevity, and Gallery of Literary and Prosperity) had a very high risk of pollen allergy, whose governance should be given the top priority (Table 5). In the 15 other spots, the number of high-risk, medium-risk, and low-risk spots were five, six, four, respectively. Among the five scenic routes, tourist risks on the two routes of the Long Corridor and Longevity Hill reached a very high or high level, while the two routes of the Rear-hill and back-lake, and East Causeway had low risks. Among the four scenic areas, both the Rear-hill and Front-hill were assessed as the high-risk area, while the risks of East-lake and West-lake areas were labeled as moderate. Basing on the above results, we drew a tourist-risk map of pollen allergy in the Summer Palace (Figure 4).

## Discussion

### Uncertainty of pollen-allergy risk under global warming and urbanization

Climate change has already brought about significant impacts on both the plant allergen and the tourist behaviors. As for the hazard of plant allergen, due to the global warming observed over the past 70 years, there have been significant changes in regional species composition (52), phenological dates (20), and ecological functions (53). These changes have subsequently affected the start, duration, and intensity of regional pollen season (21–23). A recent study estimated that under a warming scenario of SSP5 8.5, the increasing concentration of atmospheric CO_2_ would increase the likelihood of seasonal allergies throughout the continental USA, with end-of-century emissions being increased up to 200% (54). Another study from Brussels, Belgium showed that the allergy hazard in urban green spaces might increase by 11–27% in the increased allergenic potential scenarios, and by 44% in the increased pollen season duration scenario (51). In addition, precipitation and near-surface wind speed will exhibit greater regional variability in the future (55), thereby increasing the hazard uncertainty of plant allergens. When continuous precipitation occurred, it can not only affect the plant flowering, but also have a scouring effect on pollen grains, resulting in a rapid decrease in pollen concentration (56). The increase of wind speed is conductive to the pollen spread. When the wind speed reaches 4–6 m/s, the pollen quickly spreads to the downwind, resulting in more extensive pollinosis (57). Besides the above climate factors, weather and climate extremes have also shown significant regional differences (55). For example, late-spring frost risk between 1959 and 2017 increased significantly in Europe and East Asia but decreased in North America (58), indicating that differences in spring pollen emissions and duration across regions is rising. In the future, projected climate changes will still manifest differently for different weather regimes and may lead to contrasting changes in average and extreme conditions.

In regard to tourist behaviors, climate change can have a potential impact on travel decision. The Sixth IPCC Assessment Report (AR6) shows that heat waves will significantly increase the risk of morbidity and mortality, and have a profound impact on population behavior patterns and transportation choices for travel (55). David Maddison found that the number of British tourists decreased significantly with the increase of temperature (59). Another study in China suggests that the improvement on the climate comfort in high latitude and high altitude areas will be beneficial to the development of outdoor sports (60). In conclusion, as described by Robert Steiger (61), when planned excursions cannot be carried out due to climate change, travelers may make three substitutive decisions, namely alterations of the travel time, destination and activity type.

The acceleration of urbanization may not only lead to the heterogeneity warmth of inner-city areas, but also promote the intensification of air pollution and the change of local wind environment. All these potential changes are influential in uncertainties of pollen-allergy hazard. Firstly, a large temperature gradient between urban and rural will occur when the heat island effect is remarkable (62), thereby urging the flowering period and pollen season of urban allergenic plants to be earlier than those in suburbs (63). Secondly, it has been found that urban land area was significantly positively correlated with PM_2.5_ concentration (64), while the PM_2.5_ concentration can promote the increase of pollen concentration (56). Moreover, the rapid change of urban underlying surface morphology can cause the urban ventilation corridors to change (65), and changes will occur in the range and direction of pollen diffusion thereby.

To sum up, the uncertainty of pollen allergy risk in the future scenarios of global warming and urbanization may increase and subsequently pose complicated impacts on people's routine outings and leisure activities. At present, the knowledge about the impact of future climate change on the pollen allergy and tourist behaviors is very limited in China. So, it is of great significance to develop parallel observation of flowering and pollen seasons, and to do further researches on the tourist's behavior. Systematic observations and the improvement of prediction models will help us to better understand the spatial-temporal uncertainty of pollen allergy risk in the future.

### Improvement of pollen allergy risk assessment

Because of difficulties with data acquisition, we only selected nine from 18 bottom indexes in the conceptual model and utilized the easiest risk matrix to conduct the case assessment. In the future, the relevant evaluation indexes and methods can be further improved based on enriching data. On the one hand, the installation of pollen monitors and the interpretation of high-resolution remote sensing images will greatly contribute to the assessment of the comprehensive allergenicity in green spaces. In this paper, flowering duration of native allergenic plants was used as an alternative index of airborne pollen seasons. However, there are some differences between them due to the influence of atmospheric circulation (9). Therefore, the index of airborne pollen duration should be added into the follow-up assessment. Furthermore, the segment of pollen season with consideration of allergenicity difference among species is better to describe the hazard of pollen allergy than the total pollen season frequently used in previous studies (66). The calculation of a vegetation index based on high-resolution RS was proved to effectively reflect the pollen concentration at a micro scale (67), and this method should also be tried.

On the other hand, the optimization of tourist behavior investigation can make the vulnerability assessment for the pollen allergy risk more accurate. In the case study, visitors' logs data retrieved from Sina Weibo essentially reflects the activity of online tourists and is only an effective data supplement when the field monitoring data of tourists is lacking. Subsequent studies based on the mobile phone location data will better reveal the tourist exposure to the plant allergen. In fact, a case-crossover study in Belgium has already utilized uploading records from mobile medical health software to analyze the relationship between the patients' exposure to green space and their pollen allergy symptom severity (68). In addition, previous analysis showed that the prevalence of pollinosis is closely related to the age, education level and income level (35, 36). Therefore, conducting a sample survey of tourists in tourism attractions is helpful to accurately assess their human resistance to this disease.

After taking possession of more and better indexes values in the future, subjective and objective integrated weighting methods should also be tried.

## Conclusion and outlook

Climate change has already affected human health directly and indirectly in China. From the perspective of tourism management, it is imperative to assess the pollen-allergy risk of tourists and visualize it by risk mapping. For this purpose, we established an assessment index system consisting of 18 available bottom indexes, which covered three aspects of allergen hazard, tourist vulnerability and resilience of assessment units. The quantification and coupling of assessment indexes were also outlined. Taking the Summer Palace as the case study area, we obtained the values of nine representative indexes *via* phenological observation, ecological investigation, and data mining of visitors' logs on Sina Weibo. Then, we combined the I_GZA_ (Green Zone Allergenicity Index) model and three-dimensional risk assessment matrix to assess and map the risk levels of spring pollen allergy in 18 scenic spots, five scenic routes and four scenic areas. The results showed that: (1) There were 7 primary pollen-allergenic plants in the park, among which *Platycladus orientalis* and *Salix babylonica* had the highest allergenic hazard. (2) Among 18 spots, tourists faced the highest risk level of spring pollen allergy in three spots, namely the Hall of Serenity, Hall of Benevolence and Longevity, and Gallery of Literary Prosperity. (3) The two routes of the Long Corridor and Longevity Hill scored high on the pollen-allergenic risk level. (4) Among four areas, the Front-hill and Rear-hill areas were high risky for tourists.

In global warming scenarios, the allergenicity of pollen-allergy plants will be driven by the changes of climate factors, such as temperature, precipitation, wind speed, CO_2_ concentration, and extreme events. Urbanization will also affect the local climate, thermal environment and PM_2.5_ concentration. As a result of these two driving forces, greater uncertainty of pollen allergy risk may occur subsequently in the future. Therefore, under the policy background that Chinese government has placed increasing emphasis on the construction of ecological civilization and the improvement of human wellbeing and public health, the high-quality development of tourism attractions should attach importance to the risk impact of local thermal environment, climate comfort and air pollution (64, 69). The dynamic assessment of pollen-allergy risk should be institutionalized and be integrated into the evaluation of tourism experience quality. Researchers should further clarify the spatial-temporal distribution characteristics of pollen allergen and tourists in China and improve the reliability of risk assessment by optimizing the index system and assessment method. Relevant government departments should also apply the assessment results to guide decisions of green spaces planning and publish sufficient information to help pollen allergy tourists to avoid environmental triggers during the airborne pollen season.

## Data availability statement

The raw data supporting the conclusions of this article will be made available by the authors, without undue reservation.

## Author contributions

YZ processed data, constructed assessment model, and wrote the original draft. JD provided guidance for phenological observation and field survey. XL edited the draft and visualized research results. HL built the workflow framework, clarified methodology, reviewed the writing, and provided fund support. All authors have read and agreed to the published version of the manuscript.

## Funding

This research was funded by the National Natural Science Foundation of China (Grant No. 41871033) and the National Key Research and Development Program (Grant No. 2018YFA0606102).

## Conflict of interest

The authors declare that the research was conducted in the absence of any commercial or financial relationships that could be construed as a potential conflict of interest.

## Publisher's note

All claims expressed in this article are solely those of the authors and do not necessarily represent those of their affiliated organizations, or those of the publisher, the editors and the reviewers. Any product that may be evaluated in this article, or claim that may be made by its manufacturer, is not guaranteed or endorsed by the publisher.

## Footnotes

^1^ http://henan.china.com.cn/m/2021-03/29/content_41511485.html

^2^ https://www.bjnews.com.cn/detail/162356847914694.html

^3^ https://www.sohu.com/a/257639840_267106
